# Tubular ER Associates With Diacylglycerol-Rich Structures During Lipid Droplet Consumption

**DOI:** 10.3389/fcell.2020.00700

**Published:** 2020-07-29

**Authors:** Suriakarthiga Ganesan, Marjan Tavassoli, Brittney N. Shabits, Vanina Zaremberg

**Affiliations:** Department of Biological Sciences, University of Calgary, Calgary, AB, Canada

**Keywords:** diacylglycerol, budding yeast, lipid droplet, tubular ER, growth resumption

## Abstract

Growth resumption from stationary phase in *Saccharomyces cerevisiae*, is characterized by lipid droplet (LD) consumption and channeling of lipid precursors toward synthesis of membranes. We have previously determined that triacylglycerol lipolysis contributes to a pool of diacylglycerol (DAG) associated with the yeast vacuole that is enriched in structures that are in close proximity to LDs. In this study we have monitored these structures using a DAG sensor fused to GFP during isolation of LDs. A unique fraction containing the DAG sensor, with low presence of LDs, was identified. Membranes enriched in the DAG probe were obtained by immunoaffinity purification using a GFP nanobody, and the associated proteome was investigated by mass spectrometry. It was determined this LD-associated fraction was enriched in proteins known to shape the tubular endoplasmic reticulum (ER) like Yop1, Sey1, Rtn1, and Rtn2. Consistently, cells lacking three of these proteins (*rtn1*Δ *rtn2*Δ *yop1*Δ) exhibited delayed LD consumption, larger LDs and abnormal LD distribution. In addition, the triple mutant displayed aberrant localization of the DAG sensor after 5 h of growth resumption from stationary phase. Manipulation of DAG levels by overexpression of the DAG kinase Dgk1, impacted localization of the DAG probe and affected fitness of the triple mutant. Altogether these results link LD consumption to tubular ER expansion as a gateway of lipid precursors that otherwise accumulate in vacuolar associated membranes or other internal compartments. Furthermore, conversion of DAG to phosphatidic acid (PA) in the absence of a functional tubular ER was toxic to cells, suggesting the ratio of PA to DAG is critical to allow growth progression.

## Introduction

Typical growth of yeast with glucose as carbon source, is initiated with a temporary lag phase where cells reprogram their metabolism to start proliferation (Granot and Snyder, 1991, 1993). Mobilization of the neutral lipids triacylglycerols (TAGs) and sterol esters (SEs) dominates during the metabolic switch triggered by glucose. This allows cells to resume proliferation, as it provides precursors such as ergosterol, fatty acids, and diacylglycerol (DAG) for membrane lipid synthesis, which are required for secretory traffic and plasma membrane (PM) expansion (Zinser et al., 1991; Kearns et al., 1997; Zanghellini et al., 2008; Rajakumari et al., 2010; Markgraf et al., 2014; Ouahoud et al., 2018). DAG can be converted to phosphatidic acid (PA) by the action of the sole yeast DAG kinase Dgk1 (Han et al., 2008) which plays a critical role during growth re-entry from stationary phase (Fakas et al., 2011a). A balance in PA to DAG ratio regulates membrane proliferation through the Opi1 circuit which controls the expression of key enzymes in glycerolipid biosynthetic pathways mostly localized to the endoplasmic reticulum (ER; Henry et al., 2012). When cells enter stationary phase the conversion of PA to DAG by the PA phosphatase Pah1 controls TAG synthesis and lipid droplet (LDs) biogenesis (Han et al., 2006; Adeyo et al., 2011; Fakas et al., 2011b). The PA to DAG ratio also impacts other cellular processes like yeast vacuolar fusion (Sasser et al., 2012; Miner et al., 2017; Starr et al., 2019) and sporulation (Rudge et al., 1998, 2004; Liu et al., 2007).

Diacylglycerol is a key signaling lipid and intermediate in lipid metabolism. DAG is a minor component of eukaryotic membranes with lipidomic studies of wild type yeast grown in standard defined medium conditions indicating is present at 4–6 mol% of total lipids (Ejsing et al., 2009; Klose et al., 2012). Our knowledge of DAG distribution and dynamics in cell membranes is limited. DAG has the ability to spontaneously translocate bilayers on a much faster timescale than phospholipids (Bennett and Tieleman, 2012), making it challenging to be monitored with subcellular fractionation approaches, as the lipid may shift localization upon cell lysis. Therefore, investigations aimed at understanding DAG spatiotemporal distribution have taken advantage of genetically encoded chimeric constructs coupled with fluorescence microscopy. DAG−binding tandem C1 domains of several proteins including protein kinases C and D (PKC and PKD) specifically bind DAG with high affinity (Codazzi et al., 2001; Baron and Malhotra, 2002; Wang et al., 2003; Stahelin et al., 2004, 2005; Ohashi et al., 2009; Sánchez-Bautista et al., 2009). These C1 domains fused to fluorescent proteins have been widely used to monitor DAG distribution in mammalian cells and their use to monitor DAG pools in live budding yeast has been validated in recent years (Ganesan et al., 2016, 2018; Choudhary et al., 2018; Joshi et al., 2018; Romanauska and Köhler, 2018). Our group has focused on the study of cytosolic DAG pools, which display very dynamic changes during growth resumption of yeast cells from stationary phase, when LDs are consumed (Ganesan et al., 2016, 2018). Two main pools were monitored over time using DAG sensors. One pool was associated with vacuolar membranes and the other localized to sites of polarized growth. Dynamic changes in DAG distribution were observed during resumption of growth from stationary phase, when DAG is used to support phospholipid synthesis for membrane proliferation (Fakas et al., 2011a). Vacuolar membranes experienced constant morphological changes displaying DAG enriched microdomains coexisting with liquid-disordered areas. Surprisingly, the probe was hardly detected in the ER where lipid metabolic pathways consuming DAG reside. Instead, different DAG-rich structures were detected as puncta in the cytosol or in close proximity to LDs associated with vacuolar membranes (Ganesan et al., 2018). In fact, some of these structures co-purified with LDs but were not conventional LDs (Ganesan et al., 2018).

In this study we aimed to further characterize DAG-rich compartments abundant in yeast re-entering growth from stationary phase. A unique fraction, positive for the DAG sensor, with low presence of LDs was identified during LD purification steps. Membranes enriched in the DAG probe were then obtained by immunoaffinity isolation, and the associated proteome was investigated by mass spectrometry (MS). Indeed, the ER emerged as a critical compartment associated with DAG-positive membranes, with relevance for tubular ER and ER contact sites proteins being highly enriched. Evidence is presented supporting a link between DAG and tubular ER expansion during growth resumption, which acts as a gateway of lipid precursors reaching a lipid metabolic hub that supports synthesis of all lipid classes for membrane proliferation.

## Materials and Methods

### Materials

Growth medium components were purchased from MP Biomedicals. Zymolyase 100T (Cat# 120491–1) and Ficoll PM400 (Cat# 17–0300–10) were purchased from Amsbio and GE Healthcare, respectively. Nile red (ThermoFisher Cat# N1124) was diluted in DMSO as a 1 mg/ml stock. Cerulenin (Sigma # C2389) was prepared in ethanol as a 1 mg/ml stock.

### Growth Conditions, Plasmid, and Strains

Yeast strains were grown in synthetic defined minimal medium (SD: 0.67% yeast nitrogen base without amino acids, 2% glucose). Amino acids and bases were supplemented based on the yeast strain requirements. Agar (2%) was added for solid plates. Unless indicated, yeast cultures were grown at 30°C with shaking (200 rpm). Standard lithium acetate transformation protocol (Guthrie and Fink, 1991) was used and cells carrying plasmids of interest were grown on selective media. Unless otherwise indicated, for growth resumption from stationary phase, cells were grown for 48 h in defined medium, pelleted and resuspended at A_600_∼0.2–0.4 in fresh defined medium and cultured at 30°C with shaking (200 rpm) for the indicated periods of time. Cerulenin (10 μg/ml) or ethanol (0.01%) was added to wild type or *rtn1*Δ *rtn2*Δ *yop1*Δ cells at time 0 of growth resumption.

Detailed information on yeast strains and plasmids used in this study is provided in Table 1. The VPH1-mCherry::LEU2 sequence was introduced in the indicated strains as described previously (Ganesan et al., 2018).

**TABLE 1:** List of strains and plasmids used in this study.

**Strains**	**Genotype**	**Source**
BY4741	*MATa his3 leu2 met15 ura3*	Euroscarf
NDY257	*BY4741 rtn1::kanMX4 yop1::kanMX4 rtn2::kanMX4*	Voeltz et al., 2006
SEY6210.1	*MATa leu2−3,112 ura3−52 his3−*Δ*200 trp1−*Δ*901 lys2−801 suc2−*Δ*9*	Manford et al., 2012
ANDY214	*SEY6210.1 tcb1*Δ*::KANMX6 tcb2*Δ*::KANMX6 tcb3*Δ*::HISMX6*	Manford et al., 2012
Sey1-TAP	*S288C; ATCC 201388; MAT a his3*Δ*1 leu2*Δ*0 met15*Δ*0 ura3*Δ*0 SEY1-TAP::HIS3MX6*	GE Dharmacon
Yop1-TAP	*S288C; ATCC 201388; MAT a his3*Δ*1 leu2*Δ*0 met15*Δ*0 ura3*Δ*0 YOP1-TAP::HIS3MX6*	GE Dharmacon
Vph1-mCherry	BY4741 *VPH1-mCherry::LEU2*	This study
*rtn1*Δ *rtn2*Δ *yop1*Δ Vph1-mCherry	*BY4741 rtn1::kanMX4 yop1::kanMX4 rtn2::kanMX4 VPH1-mCherry::LEU2*	This study
Erg6-RFP	*MAT*α *his3*Δ*1 leu2*Δ*0 lys2*Δ*0 ura3*Δ*0 ERG6::mRFP kanMX6*	Huh et al., 2003
Rtn1-GFP	*MATa his3*Δ*1 leu2*Δ*0 met15*Δ*0 ura3*Δ*0 Rtn1-GFP:: His3MX*	Huh et al., 2003

**Plasmids**	**Description**	

pRS426-G20	*TEF2* promoter, *GFP-Spo20^51–91^ (2* μ, *URA3)*	Nakanishi et al., 2004
pGPD416-C1δ-GFP	*GPD* promoter, *C1*δ*-GFP (CEN, URA3)*	Ganesan et al., 2018
pESC *LEU2*	*GAL1/10 promoter (2* μ, *LEU2)*	Agilent
YEplac181-GAL1/10-*DGK1*	*DGK1* under control of *GAL1/10* promoter in YEplac181 (*2* μ, *LEU2*)	Han et al., 2008
YEplac181-GAL1/10-*DGK1*[D177A]	*DGK1[D177A]* under control of *GAL1/10* promoter in YEplac181 (*2* μ, *LEU2*)	Han et al., 2008

### Growth Assay

For growth curves, the A_600_ of the cultures was monitored with a Thermo Scientific^TM^ GENESYS^TM^ 30 Visible Spectrophotometer. For growth assays on solid medium, cells were grown as described above for growth resumption phase and serially diluted 1:10 beginning with A_600_∼0.5. Cells were spotted on plates containing respective synthetic defined media with either 2% glucose or galactose using a bolt replicator, and incubated at 30°C for 3 days.

### Yeast Lipid Droplet Purification and Subphase Isolation

Lipid droplets were purified as previously described (Athenstaedt, 2010) with some modifications. Briefly, yeast cells expressing the DAG sensor C1δ-GFP from a plasmid and Erg6-RFP at endogenous levels were grown for 24 h in selective drop-out media. Cultures were then diluted and allowed to grow into early stationary phase for an additional 24 h. Cells were collected without resuspension in fresh medium for stationary phase samples or resuspended in fresh medium for additional 45 min to allow growth re-entry for growth resumption phase samples. Cells were collected, washed once with water and the wet weight was determined. Cells (approximately 10 *g* of cell wet weight) were shaken for 10 min at 30°C in 0.1 M Tris/H_2_SO_4_ buffer (pH 9.4) containing 10 mM DTT (freshly added) and then washed with 1.2 M sorbitol in 20 mM KH_2_PO_4_ (pH 7.4). Zymolyase 100T (0.4 mg per gram cell wet weight) was added to obtain spheroplasts (Daum et al., 1982). Spheroplasts were washed twice with 1.2 M sorbitol in 20 mM KH_2_PO_4_ (pH 7.4) prior to homogenization. The washed spheroplasts were resuspended in buffer A [10 mM MES-Tris (pH 6.9) 12% (w/w) Ficoll 400–0.2 mM EDTA, 1 mM PMSF 1.5 μg/ml pepstatin and 1 × Complete EDTA-free protease inhibitor mixture] to a final concentration of 0.5 *g* per cell wet weight per ml. Spheroplasts were then homogenized with a Dounce homogenizer by applying 20–30 strokes using a loose fitting pistil, followed by a centrifugation at 5,000 *g* for 5 min at 4°C. The resulting supernatant (∼5 ml) was transferred into a ultracentrifugation tube and overlaid with an equal volume of buffer A, and centrifuged for 60 min in a swing-out rotor (SW40Ti) at 100,000 *g* at 4°C (Beckman Coulter Optima^TM^ L–100K). After, a white floating layer was collected from the top of the gradient and resuspended very gently in buffer A by using a homogenizer and a loose-fitting pistil. The suspension was again transferred into a ultracentrifugation tube, overlaid with buffer B [10 mM MES-Tris (pH 6.9) 8% (w/w) Ficoll 400–0.2 mM EDTA] and centrifuged for 30 min at 100,000 *g* (4°C). After the second overlay and centrifugation, tubes were allowed to rest for 30 min, and the white floating layer containing LDs was removed and suspended in buffer containing 8% (w/w) Ficoll 400/0.6 M sorbitol and overlaid with buffer containing 0.25 M sorbitol only. After 30 min of centrifugation at 100,000 *g*, a final white floating layer was collected containing highly purified LDs. The purified LDs were homogenized with 20 strokes using a Dounce homogenizer. A fraction corresponding to the bottom of the last gradient containing the final pellet (∼0.4 ml) was also collected.

After the second ultracentrifugation and removal of the top white layer, a subphase laying beneath became visible to the naked eye and was also collected (∼0.4 ml). Microscopy inspection indicated it was enriched in GFP over RFP signal. This subphase was subsequently used for C1δ-GFP membrane enrichment and proteomic analysis. The same protocol was used to isolate subphase fractions from growth resumption and stationary phase in cells expressing the C1δ-GFP plasmid and endogenously tagged Sey1-TAP or Yop1-TAP.

### Detergent-Free Immuno-Isolation of Membranes Enriched in C1δ-GFP and Mass Spectrometry Analysis

The subphase obtained above was split in two, diluted (1 ml final volume) with 1xTNE buffer containing protease inhibitors [50 mM Tris–HCl, pH 7.4, containing 150 mM NaCl, 5 mM EDTA, Complete EDTA-free protease inhibitor mixture (Roche Applied Science Cat # 5892791001), 1 mM PMSF, and 3 μg/ml pepstatin] and incubated with equilibrated agarose-beads (Biolynx Cat#VECTAG1000) or GFP-Trap (Chromotek Cat# gta-20 GFP-Trap^®^ _A) for 1 h at 4°C according to manufacturer’s indications. Beads were washed six times at 4°C with 1.0 ml sterile 1x phosphate buffered saline (PBS). All spins during the affinity isolation steps were done in a benchtop microfuge at 16,000 RCF for 30 s at 4°C to pellet the beads. Nitrile gloves and Milli-Q filtered ultrapure water were used for the immunoaffinity purification destined for MS analysis to avoid contamination with keratin. Beads were then resuspended in 40 μL of 2x gel loading buffer [GLB, 0.2 M Tris–HCl (pH 6.8), 8% SDS, 0.4% bromophenol blue, and 40% glycerol]. Samples were boiled for 10 min to elute bound proteins and were prepared for MS analysis by loading them onto a 1.0 mm, 10% SDS-PAGE gel and run just until the entire sample had entered into the resolving gel. A small square (∼1 mm^3^) of resolving gel was sliced using a razor blade. In-gel trypsin digestion and peptide analysis was performed by the Southern Alberta MS Centre at the University of Calgary. The tryptic peptides were analyzed by liquid chromatography (LC; Agilent 1260 Infinity chip cube interface) tandem mass spectrometry (MS/MS) on an Agilent 6550 iFunnel quadrupole – time-of-flight (Q-TOF) mass spectrometer. The LC and the Q-TOF were both controlled by MassHunter (version B.05.00). Tandem mass spectra were extracted by Agilent MassHunter qualitative analysis software (version B.05.00) and converted into a Mascot Generic Format file using the default parameters. For data extraction, a peptidic isotopic model with a maximum charge state of 6 was used. All MS/MS samples were analyzed using Mascot (Matrix Science, London, United Kingdom; version 2.4.0). Mascot was set up to search the NCBInr_20150121 database (selected for *Saccharomyces cerevisiae*, 50256 entries) assuming the digestion enzyme trypsin. Mascot was searched with a fragment ion mass tolerance of 0.20 Da and a parent ion tolerance of 20 PPM. Scaffold (version Scaffold_4.4.4, Proteome Software Inc., Portland, OR) was used to validate MS/MS based peptide and protein identifications.

### Microscopy

Unless otherwise indicated, all cells were mounted on 2% agarose pads made in respective growth medium on the microscopy slides. Images were acquired with a Zeiss Axio Imager Z2 upright epifluorescence microscope. ZEISS Zen blue imaging software and Zeiss plan Apochromat 100x/1.4 oil immersion objective lens were used for image acquisition. Fourteen *z*-stacks (0.4 μm) were routinely acquired and deconvolved using the constrained iterative algorithm available in the Zen software. Colibri 7 LED light and 90 High Efficiency filter sets were used for excitation of GFP and mCherry/RFP. For GFP signal, samples were excited at 470/40 nm, and emission range was set at 525/50 nm while for mCherry/RFP signal, samples were excited at 555/30 nm, and emission range was set at 592/25 nm. For simultaneous detection of Nile Red and GFP, imaging was performed using a Leica SP5 confocal microscope and a Leica HCX 63 × 1.4 NA objective. Nile Red and GFP were excited at 488 nm and GFP and Nile Red fluorescence emission was detected between 500–515 nm and 560–590 nm, respectively. All fluorescence microscopy images shown represent mid-section images.

### Nile Red Staining

Lipid droplet staining with Nile Red was performed as described previously (Wolinski and Kohlwein, 2008). In short, cells (A_600_ = 1) were collected, pelleted, and incubated with Nile Red at a final concentration of 1 μg/ml for 20 min at room temperature. Cells were then washed three times in respective growth media and imaged immediately. For stationary phase culture staining, cells were pelleted following incubation and imaged without washing to prevent growth resumption in these cells. To ensure continuous labeling, Nile Red was added at a final concentration of 1 μg/ml to the 2% agarose pads made in respective growth medium.

### Western Blot Analysis

Protein concentration was determined using the BCA assay (Thermo Scientific) with bovine serum albumin as a standard. Samples were resuspended in 1x GLB and boiled for 1 min. SDS-PAGE was carried out by the method of Laemmli (1970). Proteins were separated by 8% or 12% resolving gel containing trichloroethanol (TCE, Sigma) to visualize proteins (Ladner et al., 2004). Proteins were transferred to a polyvinylidene fluoride (PVDF) membrane (Millipore) using a Bio-Rad transfer system at 100 V for 1 h, and then stained with red Ponceau (Sigma) to confirm transfer. For western blot analysis, the following antibodies were used: monoclonal anti-GFP (Roche), monoclonal anti-Dpm1 and anti-Vma2 (Invitrogen, Thermo Fisher) as well as polyclonal antibodies raised against Pma1 and Erg6 (kind gifts from R. Serrano, Universidad Politécnica de Valencia, and G. Daum, Universität Graz, respectively) and subsequently with horseradish peroxidase-conjugated secondary antibodies (Invitrogen, Thermo Fisher). Horseradish peroxidase-conjugated rabbit anti mouse IgG secondary antibody (Millipore Sigma) was used directly to detect tandem affinity purification (TAP) tagged proteins. Enhanced chemiluminescence (Amersham, GE Healthcare) was used for detection followed by Amersham Imager 600 for visualization and imaging. Densitometry analysis for all the blots was conducted using ImageJ (Schneider et al., 2012).

### Mass Spectrometry Data and Statistical Analysis

The online clustering tool FunSpec (Robinson et al., 2002) was used to analyze protein enrichment data in the GO class cellular component. The significance score (*p*-value) associated with each cluster represents the probability that the respective list and any given functional category occurs by chance. The ClueGO plugin (Bindea et al., 2009) for Cytoscape (Shannon et al., 2003) was used to analyzed data from the metabolic KEGG database. An additional plugin, CluePedia, created maps linking the terms with their related genes. In the maps, nodes represent the enriched terms, while the edges that connect the nodes are calculated using kappa statistics (Bindea et al., 2013). In brief, the kappa score is a chance-corrected value of co-occurrence between two genes, considering both the observed and chance co-occurrence. *P*-values were calculated with the Fisher Exact Test and corrected using the Bonferroni step-down method.

### Statistical Analysis and Image Preparation

Statistical analysis for all microscopy images was performed using two−way ANOVA with Bonferroni’s post−test. To determine statistical significance, a 95% confidence interval was used. Image quantification was performed using ImageJ (NIH). GraphPad Prism 5 software was used for statistical analysis of data and preparation of figures.

## Results

### Isolation of a Lipid Droplet-Associated Fraction Containing DAG-Rich Structures

**FIGURE 1:**
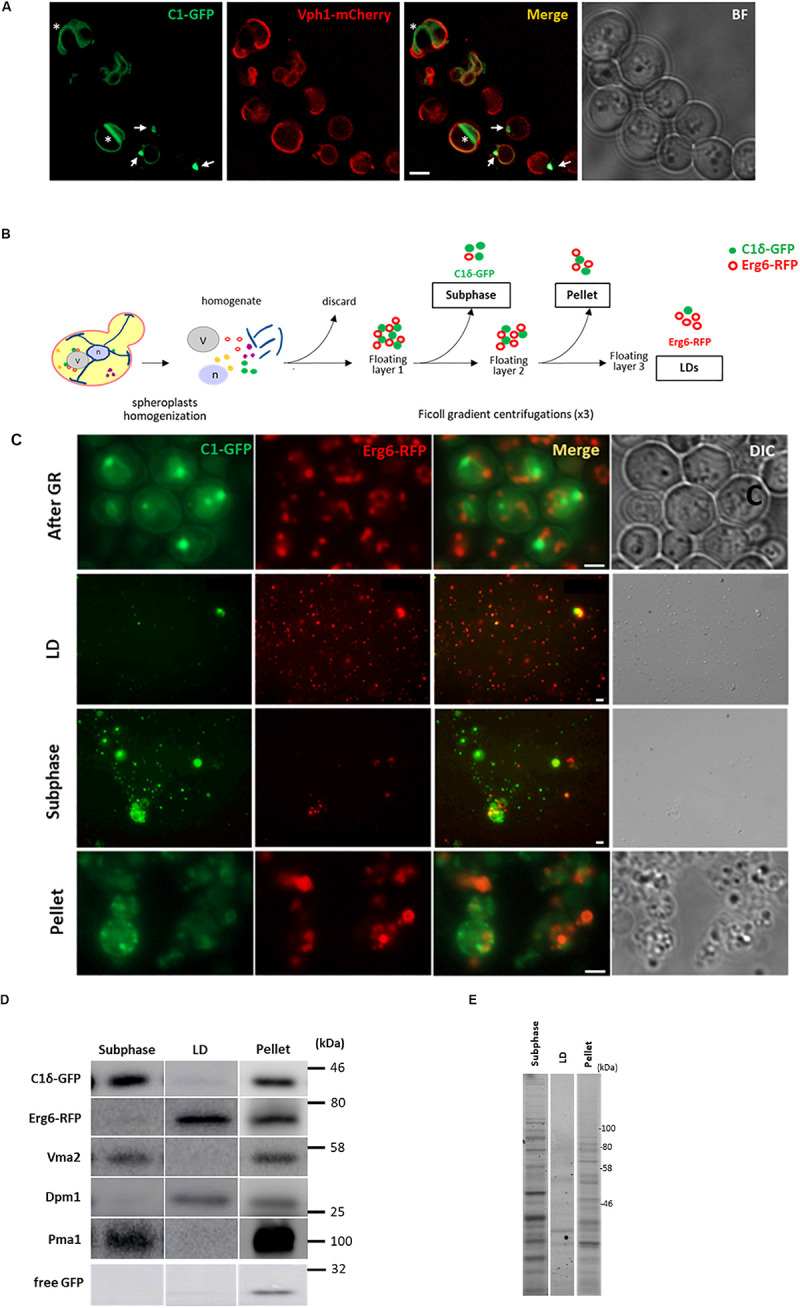
Isolation of a lipid droplet-associated fraction containing DAG-rich structures. **(A)** Wild type cells expressing the DAG sensor C1δ–GFP and endogenous vacuolar marker Vph1–mCherry were grown to stationary phase in selective medium at 30°C. Cells were then collected and resuspended in fresh media to allow growth resumption and imaged after 2 h. Asterisks and arrows point to DAG microdomains and DAG puncta associated with vacuoles, respectively. Scale bar is 2 μm. BF, brightfield. **(B)** Scheme of the approach used for lipid droplet and subphase isolation. **(C)** Representative image of cells after 45 min of growth resumption, purified LD, DAG enriched subphase and pellet fractions obtained from cells expressing the DAG sensor C1δ-GFP and endogenously expressing Erg6-RFP. Scale bars represent 1 μm. **(D)** Western blot analysis of subphase, isolated LD, and pellet fractions. Equal volumes (35 μl) were loaded for subphase and LD fractions and a third of this volume for the pellet fraction (12 μl). Expected molecular weights: C1δ–GFP (DAG sensor)—43 kDa; Erg6–RFP (LD marker)—68 kDa; Dpm1 (ER marker)—30 kDa; Vma2 (vacuole marker)—58 kDa; Pma1 (PM marker)—100 kDa; free GFP – 27 kDa. **(E)** 2,2,2-Trichloroethanol (TCE) staining showing total protein profile for subphase, isolated LD and pellet fractions.

During growth re-entry from stationary phase in yeast, abundant DAG-rich structures were seen *in vivo* associated to LDs (Figure 1A and Ganesan et al., 2018). Given this association, a strategy to isolate and characterize DAG-rich structures was devised by combining an LD purification protocol with enrichment of membranes positive for a GFP-DAG sensor. For this purpose, cells expressing the LD marker Erg6 fused to RFP and the C1δ-GFP DAG-probe (Ganesan et al., 2018) were grown to the stationary phase for 24 h in defined medium containing 2% glucose. Cells were then pelleted and resuspended in fresh medium and cultured for an additional 45 min to allow growth resumption before preparation of spheroplasts and homogenization. The LD purification protocol involved three consecutive rounds of Ficoll gradient centrifugations where a final floating white layer of LDs and a pellet comprising LD associated membranes were obtained at the end (see section “Materials and Methods” and scheme in Figure 1B). In order to identify layers containing DAG-rich structures, fractions from all gradients were methodically collected and immediately inspected under a fluorescent microscope to monitor for both GFP (DAG-rich structures) and RFP (LDs) signals. In this way, a fraction collected from the second gradient was pinpointed, displaying a dominant GFP signal with minimum detection of RFP (Figure 1C). This fraction corresponded to a buoyant subphase which detached and became visible to the naked eye, after removal of the top white floating layer in the second centrifugation. The high GFP to RFP signal in this subphase greatly contrasted with the one displayed by the LD fraction where the RFP signal dominated instead, and those from the final pellet where both GFP and RFP signals were high (Figure 1C). Western blot analysis further confirmed that the GFP signal in the subphase was indeed due to the presence of the DAG probe and was not due to free GFP resulting from its degradation as seen frequently in lysates and pellet samples (Figure 1D). Furthermore, the protein pattern of this subphase fraction was unique and different from those of the LD and pellet final fractions (Figure 1E).

**FIGURE 2:**
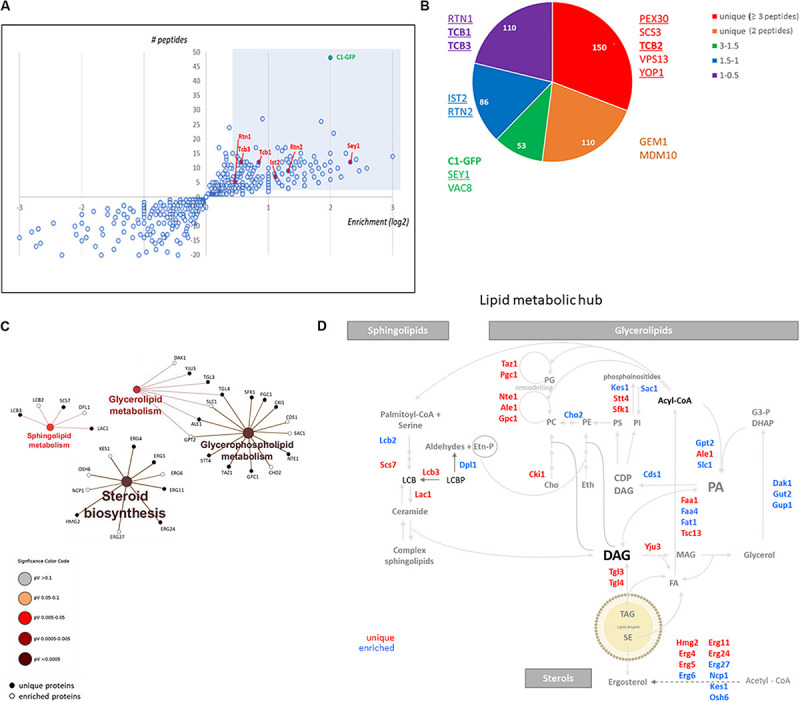
DAG enriched membranes associated with LDs during growth resumption unveil a hub for lipid metabolism. **(A)** Distribution of proteins augmented in subphase fraction. Shaded area corresponds to the cut-off used for proteomic analysis for **(B)**. **(B)** Distribution of proteins according to the categories described highlighting those associated with tubular ER and ER contact sites. Proteins identified in a previous study enriched in Yop1 are underlined. Members of the tricalbin family are in bold. **(C)** Enrichment of KEGG metabolic categories after analysis of proteins using the Cytoscape plugin ClueGo. Significance of the category nodes is indicated by their size and color code according to the scale shown at the right. Black filled circles correspond to proteins uniquely found in the GFP-trap sample, while open circles correspond to proteins enriched in C1δ-GFP membranes. **(D)** Lipid metabolic hub associated with the isolated DAG-rich membranes, including enzymatic steps in pathways for all three eukaryotic lipid classes.

To gain insight into the nature of the DAG-sensor positive structures present in the LD-derived subphase, we designed a detergent-free immunoisolation approach to obtain membranes enriched in the C1δ-GFP probe using a GFP-nanobody followed by proteomic analysis by MS. Detergents were avoided during the isolation in order to preserve protein lipid interactions and the integrity of the compartments captured. Since the GFP-nanobody was linked to agarose beads, non-specific binding of proteins from the subphase to plain agarose beads was used as control condition. Although C1δ-GFP was detected in all control samples, a 4 to 6 times enrichment was observed after the GFP-Trap step, in three independent isolations performed (Figure 2A and Supplementary Table S1), supporting the successful isolation of C1δ-GFP-enriched membranes. Interestingly, a total of 150 unique proteins not found in control samples (cut-off ≥ 3 peptides) were identified in the GFP-trap samples, in at least one round of MS analysis (Figure 2B and Supplementary Table S2). Protein localization (MIPS subcellular localization) was investigated by database search enrichment analysis and manual curation. ER (*p*-value < 1e–14), Golgi (*p*-value 1.725e–06), and mitochondrion (*p*-value 1.266e–04) were the three main organelles enriched in the set of unique proteins (70% of the proteins localizing to these compartments).

When the analysis was expanded to also include proteins enriched in the C1δ-GFP-Trap sample (2 peptides, cut-off 1.5 fold enrichment) the number of proteins increased to an additional 185 found twice and 103 found three times (Supplementary Table S1). When these additional proteins were considered (438 proteins in total) the main cellular compartments enriched were ER (*p*-value < 1e–14), mitochondria (*p*-value 9.603e-14), Golgi membrane (*p*-value 7.776e–08), and LDs (*p*-value 3.151e–06).

These results validated the isolation of a unique DAG-sensor positive fraction, enriched in membranes associated mainly with the ER and other organelles like mitochondria, Golgi, and LDs.

### DAG-Rich Membranes Unveil a Connection to Tubular ER

Since the ER emerged as one of the main compartments enriched in the isolated DAG rich membranes, we first directed our analysis to the list of ER resident proteins (85 among the 438 total proteins).

Interestingly, we noticed that this list included a large group of lipid metabolic enzymes as well as some ER structural proteins known to shape the tubular ER. Among these membrane-shaping proteins were the reticulon-interacting protein Yop1, reticulons Rtn1, and Rtn2, as well as the mammalian atlastin functional ortholog, Sey1 (Table 2; Voeltz et al., 2006; Hu et al., 2009). Remarkably, 67% of the proteins recently identified in a proteomics study of Yop1 enriched tubular ER membranes (Wang et al., 2017) were also detected in DAG rich membranes isolated in this study (53 of 79 total Yop1 associated proteins – see Supplementary Table S3). These results suggested that DAG may be abundant in the tubular ER, which is compatible with the high membrane curvature that characterizes this network (Wang et al., 2017; Ulloa et al., 2019).

**TABLE 2:** Tubular ER proteins identified in DAG positive membrane.

**Protein**	Description*
Rtn1	Reticulon protein; involved in nuclear pore assembly and maintenance of tubular ER morphology; promotes membrane curvature; regulates the ER asymmetry-induced inheritance block during ER stress; role in ER-derived peroxisomal biogenesis; increases tubular ER when overexpressed; mutants have reduced phosphatidylserine transfer between the ER and mitochondria; interacts with exocyst subunit Sec6p, Yip3p, and Sbh1p; member of the RTNLA subfamily
Rtn2	Reticulon protein; involved in nuclear pore assembly and maintenance of tubular ER morphology; promotes membrane curvature; regulates the ER asymmetry-induced inheritance block during ER stress; role in ER-derived peroxisomal biogenesis; interacts with Sec6p, Yip3p, and Sbh1p; less abundant than RTN1; member of RTNLA (reticulon-like A) subfamily; protein increases in abundance and relocalizes to plasma membrane upon DNA replication stress
Yop1	Reticulon-interacting protein; ER integral membrane protein involved in the generation of tubular ER morphology; promotes membrane curvature; forms tubules *in vitro*; regulates the ER asymmetry-induced inheritance block during ER stress; role in ER-derived peroxisomal biogenesis; interacts with Yip1p to mediate membrane traffic and with Sey1p to maintain ER morphology; facilitates lipid exchange between the ER and mitochondria; forms ER foci upon DNA replication stress
Sey1	Dynamin-like GTPase that mediates homotypic ER fusion; has a role in ER morphology; interacts physically and genetically with Yop1p and Rtn1p; functional ortholog of the human atlastin ATL1, defects in which cause a form of the human disease hereditary spastic paraplegia; homolog of Arabidopsis RHD3

*From the Saccharomyces Genome Database.

Within the group of proteins found in DAG-rich membranes that overlapped with those associated with Yop1-rich membranes were several tethering proteins known to connect the ER to the PM. Notably, the yeast tricalbin Tcb2 was part of the list of unique proteins associated with membranes enriched in the DAG probe (Supplementary Table S2 and Figure 2B). Furthermore, the other two members of this family of extended-synaptotagmins (Schulz et al., 2004) were also identified in our proteomic analysis with Tcb1 and Tcb3 peptides enriched 1.8 and 1.5 times, respectively (Figure 2B). It has been initially suggested that this family of proteins may transport DAG at ER-PM contact sites through their SMP (synaptotagmin-like, mitochondrial-lipid binding protein) domain (Saheki et al., 2016). Therefore, we next investigated the distribution of the DAG probe in cells lacking all three tricalbins but found no major alterations (Supplementary Figure S1). More recently, an alternative role for these tether proteins serving as regulatory interfaces to integrate lipid synthesis pathways rather than as physical conduits for lipid exchange at ER-PM contacts has been proposed (Quon et al., 2018). In concert with this idea, a hub of lipid metabolism was associated with the isolated DAG-rich membranes, including enzymatic steps in pathways for all three eukaryotic lipid classes, i.e., glycerolipids, sphingolipids, and sterols (Figures 2C,D). Specifically, sphingoid base metabolism and ceramide synthesis, but not complex sphingolipids metabolic steps were represented. Ergosterol synthesis and transport by Kes1 (Osh4) and Osh6 were identified. Kes1 is a sterol/phosphatidylinositol-4-phosphate [PI(4)P] exchanger (de Saint-Jean et al., 2011). Interestingly, PI(4)P kinases Stt4 and Sfk1 as well as the PIP phosphatase Sac1 were also identified in DAG-rich membranes.

Crosstalk between the sphingolipid and PI(4)P pathways occurs through a conserved multiprotein complex that includes the serine palmitoyltransferase (Lcb1 and Lcb2), Orm1, and Orm2, Tsc3, and Sac1 (SPOTS; Breslow et al., 2010). Indeed, three members of the complex were identified in the isolated DAG-rich membranes including Lcb2, Orm2, and Sac1.

Therefore, these results point to tubular ER and ER contact sites as DAG-rich compartments involved in the regulation of flow of lipid precursors toward all major lipid biosynthetic pathways. Rate limiting steps catalyzed by Lcb2 (sphingolipids), Gpt2, and Cds1 (glycerophospholipids) and Hmg2 (sterols) all converged in these DAG-rich membranes. Since our experiments were conducted with cells re-entering growth from stationary phase, the specific relevance of the tubular ER during this growth period was next investigated.

### Tubular ER Expansion During Growth Resumption Affects Cytosolic DAG Pools and Efficient Lipid Droplet Consumption

**FIGURE 3:**
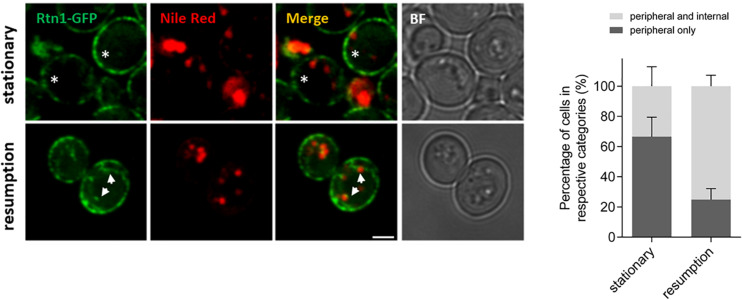
Tubular ER expands upon growth resumption from stationary phase. Cells were grown in defined medium for 48 h into stationary phase, imaged immediately (stationary) or diluted in fresh medium, and imaged after 2 h (growth resumption) using confocal microscopy. Cells were incubated with Nile Red as described in Materials and Methods. Asterisks and arrows point to peripheral and internal signal, respectively. Scale bar is 3 μm. BF, brightfield **(left)**. Quantification for localization of Rtn1-GFP in stationary and during growth resumption. Data shown are representative means ± SD for three independent experiments (*n* = total of 196 cells were scored for each growth condition). Statistical analysis was performed using two-way ANOVA where ^∗∗^*P* < 0.0001 **(right)**.

The role of ER-shaping proteins and the tubular ER network on LD consumption and distribution of cytosolic DAG pools during re-entry of growth was further investigated. In order to examine the localization of the tubular ER with respect to LDs, cells expressing endogenous levels of Rtn1 fused to GFP (tubular ER marker) were stained with Nile Red to localize LDs in both stationary phase or after 2 h of growth resumption. Inspection of cells with confocal microscopy indicated that the tubular ER network expanded from a dominant peripheral localization in stationary phase to the interior of the cell upon re-entry of growth (33 vs 75% intracellular signal in stationary and GR, respectively; Figure 3). Consistently, points of contact between LDs and the tubular ER network were observed.

**FIGURE 4:**
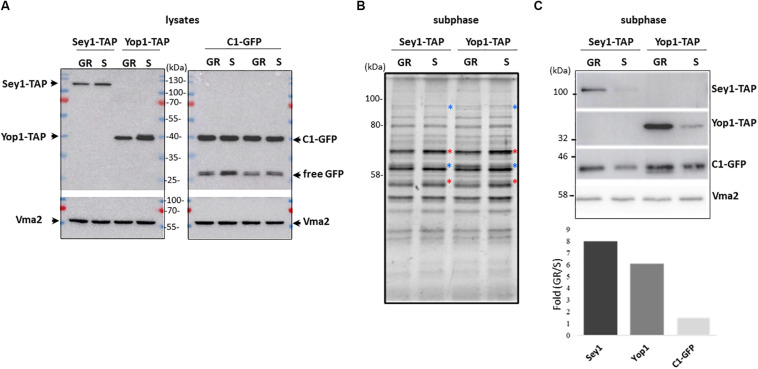
Association of the ER-shaping proteins Yop1 and Sey1 with the LD-associated subphase increases upon growth resumption. **(A)** Twenty micrograms of total protein from the indicated whole cell lysates were loaded in each lane, transferred to a PVDF membrane, and blotted to detect the indicated proteins. **(B)** Protein profile as observed with 2,2,2-Trichloroethanol (TCE) staining of the LD-associated subphase fractions (before GFP enrichment). **(A)** 1.5% of the total isolated fraction was loaded in each lane. Red and blue asterisks point to bands that decreased and increased during growth resumption compared to stationary phase, respectively, **(C)** Western blot analysis of the gel shown in **(B)** and densitometry of the bands in the bottom. Quantification is expressed as fold increase upon growth resumption compared to stationary phase for Sey1-TAP, Yop1-TAP, and C1-GFP signal shown in **(C)** normalized using Vma2 as loading control. Quantified values for C1-GFP were the same for both independent Sey1-TAP and Yop1-TAP samples. Rabbit anti mouse IgG crosslinked to horse radish peroxidase was used to detect protein A-tagged Yop1 and Sey1. Expected molecular weights: Yop1-TAP – 41 kDa; Sey1-TAP – 110 kDa, C1δ-GFP (DAG sensor)—43 kDa; Vma2 (vacuole marker)—57 kDa; and free GFP – 27 kDa.

With the premise that during growth re-entry DAG levels increase due to LD consumption with a concomitant tubular ER expansion, we decided to analyze the levels of ER shaping proteins Yop1 and Sey1 in LD-associated subphases from cells in both stationary and resumption phases of growth. For this purpose, the DAG probe was expressed in two independent strains in which Yop1 or Sey1 were tagged with a TAP tag to allow their visualization via western blot (Figure 4). After subphase isolation, the total protein profile of the two sets of independent samples were compared (Figure 4B). Clear differences were observed based on the phase of growth. While the abundance of some proteins increased during growth resumption others decreased compared to samples from stationary phase, suggesting this LD-associated fraction captures changes associated with the phase of growth. We identified this LD-associated subphase as a fraction with tubular ER proteins specifically detected after further enrichment of membranes positive for the DAG probe. Since DAG structures are more abundant during re-entry of growth, we next examined if this was reflected in the levels of the C1δ-GFP probe as well as tubular ER proteins Yop1 and Sey1 in samples obtained from cells that resumed growth. Indeed, the abundance of all three proteins in this fraction increased during growth resumption compared to Vma2 which showed no changes and was therefore used as a control for densitometric analysis (Figure 4C). While Sey1 and Yop1 proteins were 8 and 6 times enriched, respectively, C1δ-GFP only increased 1.5 times (Figure 4C bottom). Importantly, these differences were not observed in whole cell lysates (Figure 4A). These results further support our findings from the unbiased proteomics analysis unveiling a new connection between LD consumption leading to increase DAG levels and tubular ER expansion. The impact of the tubular ER on cytosolic DAG pools was examined next.

**FIGURE 5:**
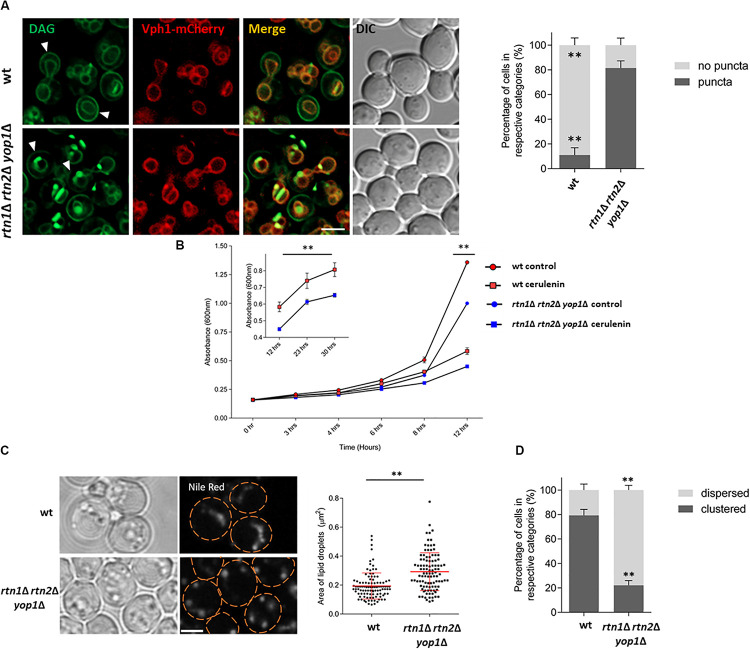
Lack of tubular ER affects DAG localization and efficient LD consumption **(A)** Wild type and the tubular ER mutant *rtn1*Δ *rtn2*Δ *yop1*Δ expressing C1δ-GFP and the endogenous vacuolar marker Vph1-mCherry were grown for 48 h in selective medium at 30°C and resuspended in fresh medium to allow growth resumption. Images were captured 5 h after cells resumed growth. Arrows point to polarized C1-GFP signal at the PM of bud cells. Scale bar is 3 μm (left). Cells from experiments in left were scored for the presence of DAG puncta. Data shown are representative means ± SD for one of the three independent experiments, (*n* = total of 223 cells were scored for each strain). ^∗∗^*P* < 0.0001 (right). **(B)** Wild type or *rtn1*Δ *rtn2*Δ *yop1*Δ cells expressing the DAG sensor C1δ–GFP were grown to stationary phase for 48 h, and then allowed to re–enter growth for 5 h in the presence or absence of 10 μg/ml cerulenin and growth (A_600_) was monitored at indicated time points. Each data point represents the average of three independent cultures. Inset highlights growth curve from 12 to 30 h. From 12 h and onward, all the treatments are significantly different based on two-way ANOWA followed by Tukey’s multiple comparison analysis. ^∗∗^*P* < 0.0001. **(C)** Cells were grown as in **(B)** for 48 h and then diluted in fresh media and imaged after 5 h. Cells were incubated with Nile Red (1 μg/ml) for 20 min before imaging to visualize LDs. Scale bar is 3 μm (left). Size of lipid droplets were quantified for **(C)** (*n* > 100) and shown as a box plot. Each spot represents a single lipid droplet. Data shown are representative means ± SD for one of the two independent experiments. Statistical analysis was performed using two-tailed student’s *t*-test where ^∗∗^*P* < 0.0001 (right). **(D)** LD distribution for cells in **(C)** was quantified (*n* = total of 196 cells were scored for each). ^∗∗^*P* < 0.0001. Data shown are representative means ± SD for one of the two independent experiments performed.

For this purpose, the DAG sensor C1δ-GFP was expressed in cells lacking the ER-shaping proteins Rtn1, Rtn2, and Yop1. This triple mutant has disrupted peripheral ER structures due to a decreased tubular ER formation (Voeltz et al., 2006). DAG localization was then monitored 5 h after cells from stationary phase were resuspended in fresh medium. Interestingly, an abnormal accumulation of DAG-rich structures was observed in the triple *rtn1*Δ *rtn2*Δ *yop1*Δ mutant, with a 70% increase in the abnormal distribution of the probe compared to the wt (Figure 5A). In addition, the polarized distribution of DAG at the PM of buds was concomitantly reduced compared to the wt. These results suggest a role for the tubular ER in regulating DAG distribution due to DAG channeling into metabolic pathways localized to the ER, or movement to other cellular compartments were this could happen.

The probe accumulation displayed by the *rtn1*Δ *rtn2*Δ *yop1*Δ mutant defective in tubular ER, was similar to the one seen in cells lacking the DAG kinase Dgk1 (*dgk1*Δ) treated with the fatty acid synthesis inhibitor cerulenin (Ganesan et al., 2018). In the presence of cerulenin LD mobilization is enhanced, as under this condition cells rely almost exclusively on TAG lipolysis as the cellular source of fatty acids to resume growth. Treatment of *rtn1*Δ *rtn2*Δ *yop1*Δ with cerulenin resulted in growth being slightly delayed (Figure 5B) and cells displaying larger LDs (Figure 5C), pointing to a retardation in TAG mobilization. Interestingly, while LDs were frequently clustered in wild type cells, mutant *rtn1*Δ *rtn2*Δ *yop1*Δ cells displayed a more dispersed LD distribution (Figure 5D). This abnormal LD phenotype did not depend on the presence of cerulenin (Supplementary Figure S2). These findings suggest that the tubular ER is involved in LD positioning and efficient mobilization of neutral lipids, impacting DAG distribution and its proper channeling for membrane lipid biosynthesis.

**FIGURE 6:**
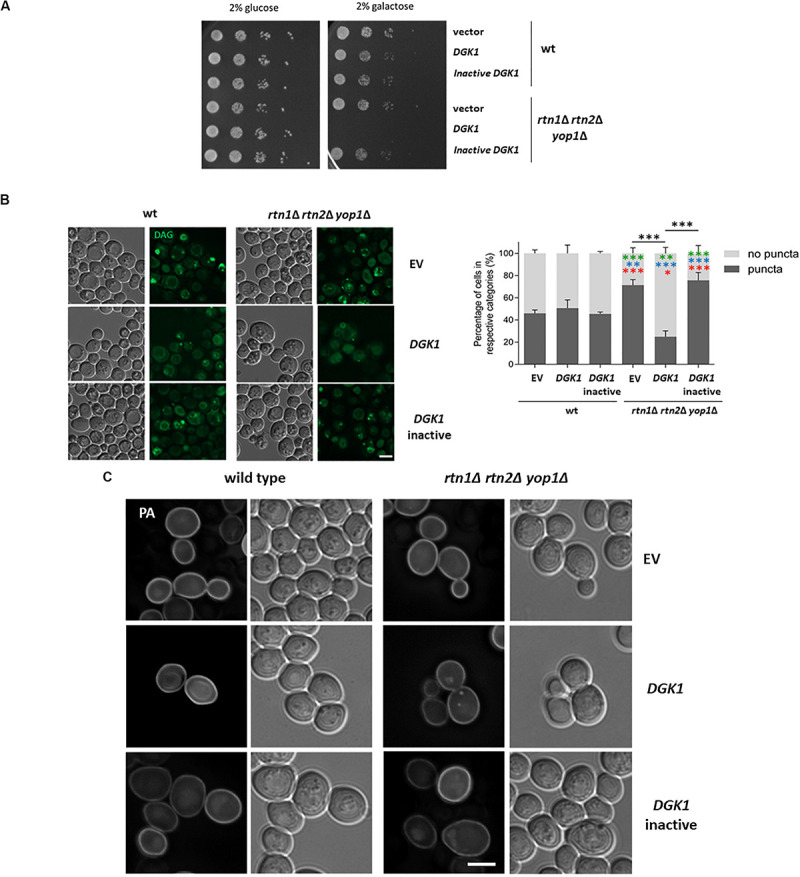
Tubular ER proteins are required for proper channeling of DAG upon growth resumption **(A)** Wild type and the tubular ER mutant *rtn1*Δ *rtn2*Δ *yop1*Δ expressing *DGK1* were grown to stationary phase and resuspended in fresh medium to allow growth resumption. Cells were plated on defined medium with glucose (control) or galactose to allow expression of genes under *GAL* promoter and plates were imaged after 2 days. **(B)** Wild type and *rtn1*Δ *rtn2*Δ *yop1*Δ yeast transformed with either empty vector (EV), *DGK1* or catalytically inactive *DGK1*^D177A^ containing plasmids (*LEU2*) as well as the DAG sensor C1-GFP plasmid (*URA3*) were grown for 48 h in defined medium containing 2% raffinose, allowed to resume growth in defined medium containing 2% galactose to induce *DGK1* expression and imaged after 2 h. Scale bar is 3 μm (left). Cells were scored for the presence of DAG puncta. Data shown represent one of two independent experiments performed. *n* = at least 77 cells were scored for each transformant except for *rtn1*Δ *rtn2*Δ *yop1*Δ expressing catalytically active *DGK1* due to cell death (*n* = 48). Green, blue and red asterisks show statistical comparison of the mutant transformants to wt (EV), wt (*DGK1*), and wt (inactive *DGK1*^D177A^), respectively. ^∗∗∗^*P* < 0.0001; ^∗∗^*P* < 0.001^∗∗^; and *P* < 0.01^∗^ (right) **(C)** Wild type and the tubular ER mutant *rtn1*Δ *rtn2*Δ *yop1*Δ expressing GFP-Spo20 and overexpressing empty vector, *DGK1* or catalytically inactive *DGK1*^D177A^ were grown and imaged as in B. Scale bar is 3 μm.

One possible scenario is that during lipolysis, LDs could be linked through DAG enriched membranes to the tubular ER, allowing conversion of DAG to PA via Dgk1. Therefore, defects in tubular ER could partially resemble *dgk1*Δ phenotypes. In fact, negative genetic interactions have been reported in global studies between *dgk1*Δ and the tubular ER mutants *rtn1*Δ, *sey1*Δ and *scs2*Δ (Schuldiner et al., 2005). It has been previously shown that overexpression of Dgk1 results in nuclear envelope expansion and accumulation of PA in the perinuclear ER (Han et al., 2008). Therefore, we tested if overexpression of *DGK1* could revert the abnormal distribution of the cytosolic pools of DAG in the tubular ER mutant *rtn1*Δ *rtn2*Δ *yop1*Δ, improving the delayed growth resumption phenotype. To our surprise, overexpressing *DGK1* in the triple mutant caused a severe growth deficiency (Figure 6A). This phenotype was dependent on the conversion of DAG to PA, as this phenotype was not observed when cells overexpressed a catalytically inactive Dgk1^D177A^ (Han et al., 2008). Inspection of transformants expressing the DAG probe confirmed that overexpression of *DGK1* reverted the accumulation of DAG-rich structures in the tubular ER mutant *rtn1*Δ *rtn2*Δ *yop1*Δ (25% vs 71% in *rtn1*Δ *rtn2*Δ *yop1*Δ expressing *DGK1* or empty vector, respectively) and this was dependent on its catalytic activity (Figure 6B). Therefore, we next decided to also monitor PA pools in the transformants using the GFP−Spo20^51–91^ probe (Nakanishi et al., 2004). As previously observed, overexpression of *DGK1* in wild type cells resulted in the probe being recruited to an enlarged perinuclear ER, in addition to its typical localization to the cell periphery. In great contrast, PA puncta accumulated in the tubular ER mutant *rtn1*Δ *rtn2*Δ *yop1*Δ overproducing catalytically active Dgk1 (21 vs 0% in *rtn1*Δ *rtn2*Δ *yop1*Δ expressing *DGK1* or empty vector, respectively; Figure 6C). These results point to a toxic effect of PA in the absence of ER-shaping proteins, and further highlight the role of the tubular ER in the proper channeling of DAG during LD consumption.

## Discussion

The present study was designed to obtain information on the nature of DAG-rich structures observed in living yeast cells. Since cells resuming growth from stationary phase display abundant DAG-rich structures due to LD consumption (Ganesan et al., 2018) we used this condition to successfully capture these compartments with a sensitive DAG sensor during LD purification. This approach led to the identification of an LD-associated subphase enriched in DAG-sensor positive membranes. Proteomic analysis of isolated DAG-rich structures identified the tubular ER as an enriched compartment in this fraction (Figure 2B and Table 2). Other organelles enriched were mitochondria, Golgi and LDs, which can communicate with the ER through membrane contact sites (MCSs; Eisenberg-Bord et al., 2016; Jain and Holthuis, 2017). The ER is the main supplier of lipids and their transport to all cellular compartments is facilitated by the MCSs. A significant number of tethering and lipid transport proteins involved in mediating or localizing to MCSs between ER-mitochondria, ER-Golgi, ER-PM, ER-LDs, and ER-vacuole were detected in the subphase and were particularly enriched in isolated DAG-rich membranes (Supplementary Tables S1, S2). Mobilization of neutral lipids stored in LDs upon growth resumption from stationary phase would result in a local increase in the levels of DAG, sterols and fatty acids. Build-up of a DAG pool dependent on TAG lipolysis has been detected at vacuolar membranes and cytosolic puncta during the first hours of re-entry of growth (Ganesan et al., 2018). An insight on how DAG would impact the interaction of LDs with bilayers could be considered from studies investigating LD emergence. It has been proposed that during LD biogenesis in the ER, an increase in the membrane DAG would facilitate formation of embedded LDs, while for transition of the generated LDs to the emerged state, the amount of DAG in the membrane must be decreased (Choudhary et al., 2018). The reverse process could be happening during LD consumption. In this scenario, DAG accumulation would induce LDs to become embedded in the membrane, allowing flux of lipid precursors directly into lipid biosynthetic pathways, while TAG synthesizing enzymes would be inhibited, preventing a futile cycle (Kiegerl et al., 2019).

Large concentrations of DAG would introduce curvature and could destabilize bilayers, serving as cue for the recruitment of proteins. For example, it has been shown that membrane recruitment and activity of Arf1 GTPase-activating protein (Arf1GAP) in mammalian cells requires DAG (Antonny et al., 1997). Arf1GAP is a protein with an amphipathic lipid packing sensor (ALPS) motif that can recognize packing defects in membranes. Proteins containing this motif were shown to bind membranes rich in DAG or with high curvature (Antonny et al., 1997; Vanni et al., 2013). Our study indeed identified several ALPS-containing proteins enriched in isolated DAG-rich membranes (Supplementary Table S4). Among these were Gcs1, an ArfGAP involved in ER-Golgi transport, and the lipid transfer proteins Kes1 and Vps13 localized to various MCSs (Antonny et al., 1997; Doucet et al., 2015). Interestingly, both Gcs1 and Kes1 contribute to DAG homeostasis in the Golgi in a process dependent on the lipid transfer protein Sec14 (Fang et al., 1996; Li et al., 2002; Yanagisawa et al., 2002). The ability of Gcs1 to bind to membranes highly curved containing DAG is essential in the Sec14-controlled circuit, where Sec14 promotes the formation of DAG at the expense of PC (Antonny et al., 1997). It has been suggested Kes1 could counterbalance Sec14 activity by sensing defects in membrane packing and modifying the sterol content to regulate Gcs1 recruitment (Drin et al., 2007). Kes1 activity involves sterol/PI(4)P exchange, followed by Sac1−dependent turnover of this phosphoinositide at the ER to produce PI. Interestingly, the PI(4)P phosphatase Sac1 was also highly enriched in DAG-positive membranes isolated in this study. It has been shown that elevated PI(4)P due to *SAC1* deletion, inhibits LD utilization and this is controlled by the Sec14-like protein Pdr16 (Ren et al., 2014; Teixeira et al., 2018), which was likewise identified herein. Therefore, we are tempted to speculate that DAG produced during LD consumption may play an important role in the recruitment of proteins like Gcs1, Kes1, and Sac1 to participate in a Pdr16-regulated circuit to control LD homeostasis. This should be addressed in future studies.

An important ER-associated lipid metabolic hub including enzymes of pathways for biosynthesis of the three lipid classes (glycerolipids, sphingolipids and sterols) was associated with the DAG enriched subphase. Interestingly, Gpt2, and Lcb2 catalyzing the rate limiting steps in the biosynthesis of PA and ceramide, respectively, were identified in our proteomic approach characterizing DAG-rich membranes. These two enzymes also have predicted ALPS motifs (Drin et al., 2007) opening the possibility they could sense membranes highly curved by DAG. It is worth noting Lcb2, Sac1, and the regulatory protein Orm2 also found in our study are part of the SPOTS complex that coordinates lipid metabolism in response to nutrients.

It has been shown, that regulation of the glycerol-3 phosphate acyltransferase (GPAT) Gpt2 through phosphorylation prevents a futile cycle during LD consumption (Kiegerl et al., 2019). Interestingly, the serine residues phosphorylated in this GPAT are part of the predicted ALPS motif. Evidence from our group indicates that lack of phosphorylation at the ALPS in Gpt2 impacts the proteome of the subphase and LDs (our unpublished results) resulting in aberrant DAG distribution during growth re-entry from stationary phase. Furthermore, Gpt2 associates with LDs forming unique ER crescent structures (Marr et al., 2012) and has been consistently found in membranes containing the ER-shaping proteins Sey1 and Yop1 (Wang et al., 2017 and our unpublished results). The role of this GPAT and its ALPS motif on DAG metabolism and tubular ER function during growth resumption is currently being investigated in our group.

**FIGURE 7:**
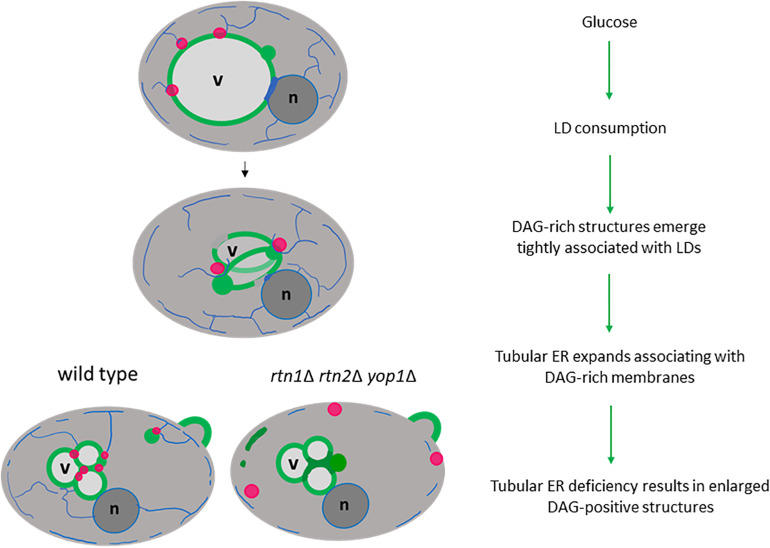
Tubular ER associates with DAG-rich structures during LD consumption. Glucose induces a metabolic switch where yeast cells resuming growth from stationary phase start consuming LDs (magenta circles) to allow membrane proliferation. During this period DAG-rich structures (green circles) emerge in tight association with LDs. An expansion of tubular ER (blue network) allows DAG to flow in wild type cells, but the DAG-rich structures are enlarged in cells defective in tubular ER shaping proteins (*rtn1*Δ *rtn2*Δ *yop1*Δ). Therefore, defective tubular ER impacts DAG distribution and the efficiency of LD consumption. Vacuole, v; nucleus, n.

Our study strongly supports a role for the tubular ER as a critical platform that connects LD consumption with the concomitant elevation of the conical lipid DAG, altering membrane curvature for the recruitment of proteins that promote membrane proliferation in the presence of glucose (Figure 7). Interestingly, a study in sea urchin eggs demonstrated that depletion of DAG levels leads to increased ER sheets-like structures at the expense of tubular ER formation (Domart et al., 2012), supporting the idea that DAG could be involved in the recruitment of ER-shaping proteins, allowing this lipid precursor to flow into ER metabolic pathways. In fact, we show herein, that defective tubular ER impacts DAG distribution and the efficiency of LD consumption.

The lethality of *rtn1*Δ *rtn2*Δ *yop1*Δ mutant cells overexpressing *DGK1* is intriguing. The fact that this phenotype depends on Dgk1 catalytic activity points to PA accumulation being toxic. In line with our findings, it has been previously shown that cells lacking the protein phosphatase Nem1/Spo7 are synthetic lethal with *rtn1*Δ *yop1*Δ (Dawson et al., 2009). Nem1 and Spo7 are the catalytic and regulatory subunits, respectively, of the ER protein phosphatase complex that regulates the PA phosphatase Pah1 (Karanasios et al., 2013). In the absence of Nem1 or Spo7, PA levels increased resulting in enlarged perinuclear ER, similarly to cells overexpressing *DGK1* (Han et al., 2008). Mutant cells *spo7*Δ and *nem1*Δ also have impaired sporulation and it was speculated that normal nuclear morphology and nuclear pore complex (NPC) assembly could be a prerequisite for meiotic nuclear division (Siniossoglou et al., 1998). It was indeed later shown that *rtn1*Δ *yop1*Δ cells display abnormal NPC distribution (Dawson et al., 2009). Therefore, the lethal effect of *DGK1* overexpression in cells lacking tubular ER could be due to an effect on NPC assembly/distribution. It is worth mentioning that several proteins of the NPC were uniquely found in isolated DAG-rich membranes, including Nup157, Nup170, Nup188, and Nup192 (Supplementary Table S2). Interestingly, both Nup170 and Nup188 have predicted ALPS motifs (Drin et al., 2007), making them good candidates to sense highly curved membranes due to DAG accumulation during re-entry of growth, to coordinate NPC assembly and membrane proliferation. Future studies should investigate this possible mechanism and the impact of the PA/DAG ratio in its regulation.

## Data Availability Statement

All datasets presented in this study are included in the article/Supplementary Material.

## Author Contributions

All authors participated in the experimental design, performed the experiments, prepared the figures, and discussed the results. SG and VZ wrote the manuscript.

## Conflict of Interest

The authors declare that the research was conducted in the absence of any commercial or financial relationships that could be construed as a potential conflict of interest.

## Supplementary Material

FIGURE S1 Cells lacking Tcb1, Tcb2, and Tcb3 display normal DAG distribution. Representative images of wt and *tcb1*Δ *tcb2*Δ *tcb3*Δ cells expressing C1δ-GFP during log phase. Scale bar is 2 μm.

FIGURE S2 Dispersed LD distribution in cells lacking tubular ER. Wild type and cells lacking Rtn1, Rtn2, and Yop1 were grown to stationary phase for 48 h in synthetic defined medium and then diluted in fresh medium and imaged after 5 h. Cells were incubated for 20 min with 1 μg/ml of Nile Red before imaging. Scale bar represents 2 μm.

TABLE S1 Peptide identification results for proteins identified either as unique or enriched twice or thrice (289 total) in DAG rich subphase.

TABLE S2 Peptide identification results for unique proteins during first round to characterize DAG rich subphase fraction (3 peptides or more).

TABLE S3 Comparison with Yop1 proteome (Wang et al., 2017, eLife).

TABLE S4 ALPS containing proteins identified in the LD-associated subphase and DAG-rich compartments.
