# Supplementary figures and images for: Tubular ER Associates With Diacylglycerol-Rich Structures During Lipid Droplet Consumption
Source: Front Cell Dev Biol. 2020 Jul 29;8:700. doi: 10.3389/fcell.2020.00700 (PMC7403446; doi:10.3389/fcell.2020.00700)

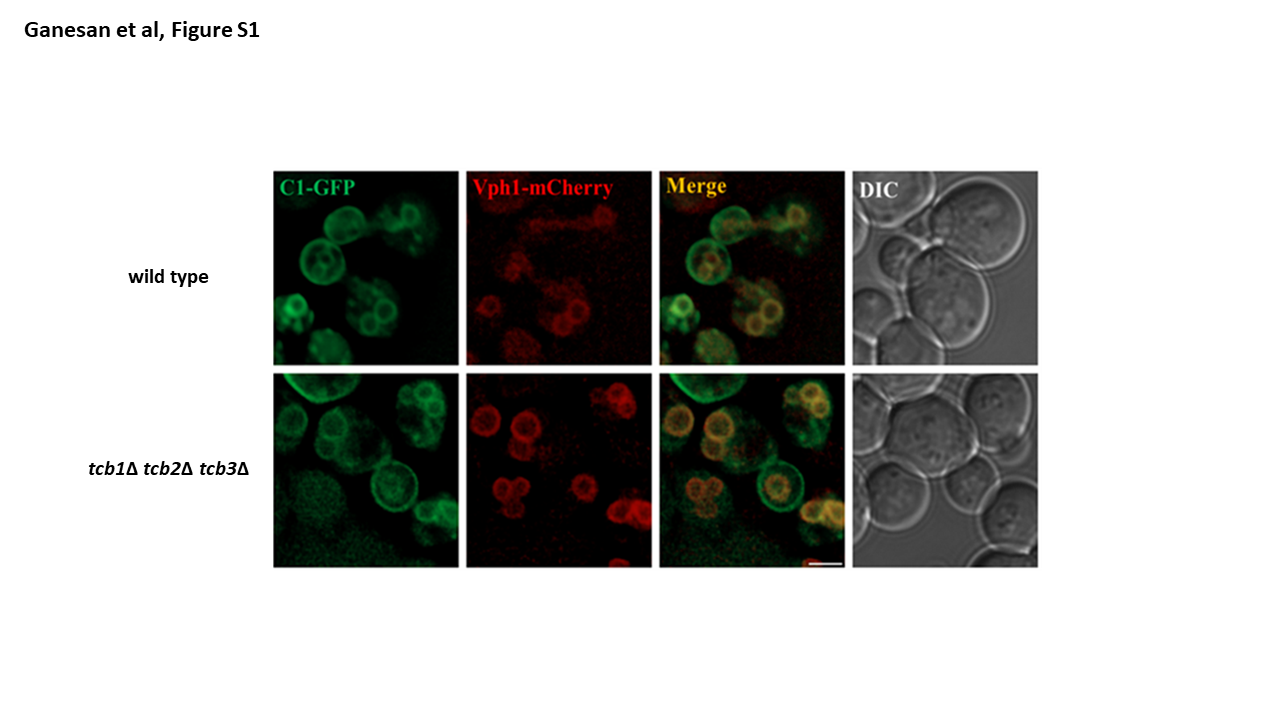

Supplement: FIGURE S1 — Cells lacking Tcb1, Tcb2, and Tcb3 display normal DAG distribution. Representative images of wt and tcb1Δ tcb2Δ tcb3Δ cells expressing C1δ-GFP during log phase. Scale bar is 2 μm. [file Image_1.tif]

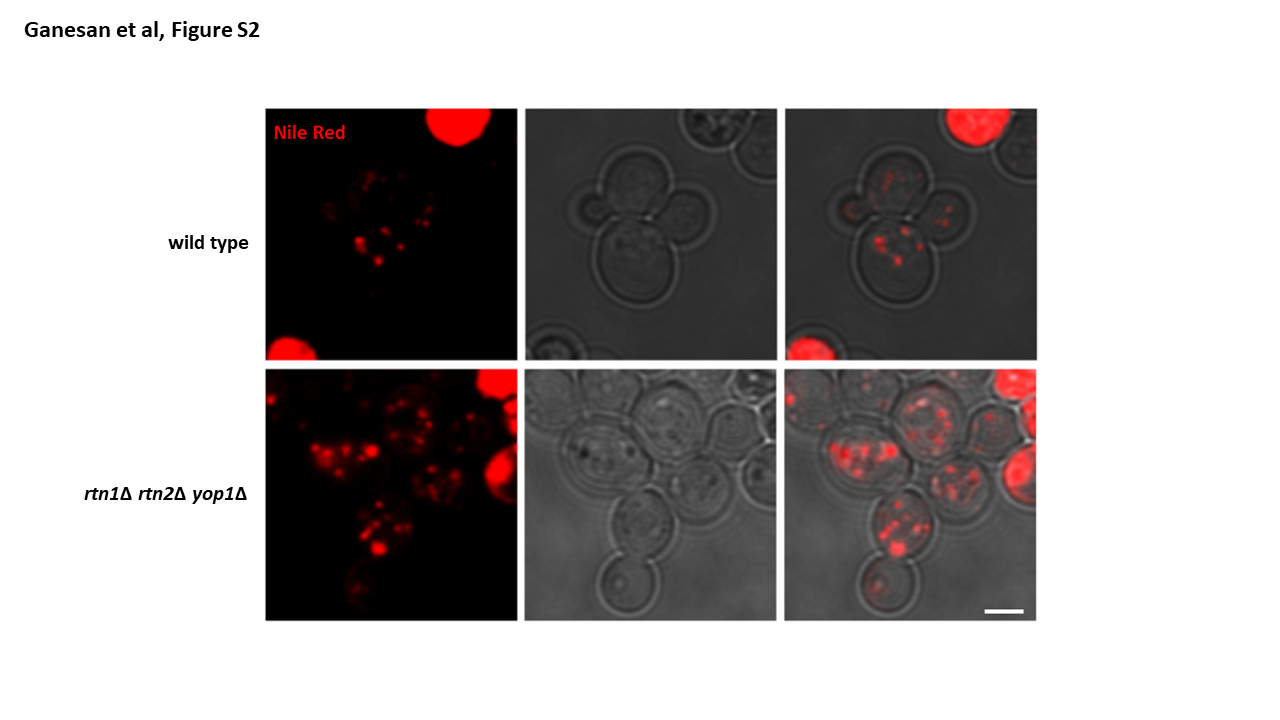

Supplement: FIGURE S2 — Dispersed LD distribution in cells lacking tubular ER. Wild type and cells lacking Rtn1, Rtn2, and Yop1 were grown to stationary phase for 48 h in synthetic defined medium and then diluted in fresh medium and imaged after 5 h. Cells were incubated for 20 min with 1 μg/ml of Nile Red before imaging. Scale bar represents 2 μm. [file Image_2.tif]
